# The first *Strigocis* Dury (Coleoptera, Ciidae) from the southern Neotropical region and a provisional key to world species

**DOI:** 10.3897/zookeys.81.940

**Published:** 2011-02-18

**Authors:** Cristiano Lopes-Andrade

**Affiliations:** Departamento de Biologia Animal, Universidade Federal de Viçosa, 36570-000, Viçosa, Minas Gerais, Brazil

**Keywords:** Minute tree-fungus beetles, Ciinae, new species, southeastern Brazil

## Abstract

*Strigocis vicosensis* Lopes-Andrade, **sp. n.** is described based on specimens from a single locality (type locality: Viçosa, state of Minas Gerais, southeastern Brazil), being the southernmost record of a *Strigocis* Dury species. Diagnostic to this new species are the dorsal vestiture consisting of stout yellowish bristles, pronotal punctures separated from each other by at least 0.75× puncture-width and sutural flange of elytra not diverging near apex. Males have both frontoclypeal ridge and anterior pronotal margin produced forward and emarginated at middle forming two small subtriangular plates, and a small abdominal sex patch. Among the New World *Strigocis*, it most resembles *Strigocis bilimeki* (Reitter), of which images of male terminalia, as far as ventral and dorsal SEM images of males are also provided. The morphological limits of *Strigocis* are briefly discussed and redefined, and a provisional key to the world species is provided.

## Introduction

*Strigocis* Dury (Coleoptera: Ciidae: Ciinae) comprises five described species (sensu Lawrence 1971): *Strigocis bicornis* (Mellié) from central and southern Europe, *Strigocis bilimeki* (Reitter) from Mexico and probably occurring in southern Arizona, *Strigocis opacicollis* Dury and *Strigocis opalescens* (Casey) from eastern North America and Mexico, and *Strigocis tokunagai* (Nobuchi) from Hokkaido, Japan. Therefore, the known species occur in the Nearctic, northern Neotropical and Palearctic regions.

In the latter decade, small series of a new *Strigocis* were collect at Viçosa, in the state of Minas Gerais, southeastern Brazil. In spite of the great collection effort in several states of Brazil, the species was not found in any other locality. Here, I describe it as *Strigocis vicosensis* sp. n., compare to the other described *Strigocis* and provide a provisional key to the world species of the genus.

## Material and methods

**Figures 1–3.**
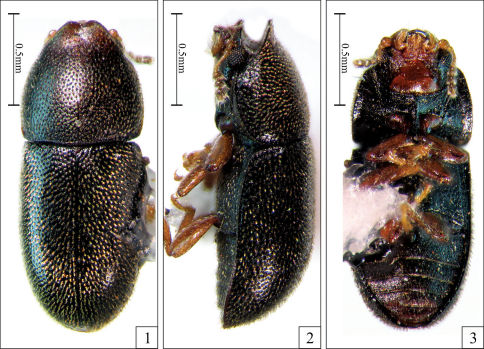
*Strigocis vicosensis* Lopes-Andrade, sp. n., adult male holotype. **1** Dorsal view **2** Lateral view **3** Ventral view.

Measurements, final comparisons and description of general external morphology were made under a Zeiss Stemi 2000 stereomicroscope with a scale ocular. Digital photographs of the holotype were taken with a Canon S70 adapted to a Leica MZ16 stereomicroscope, and final images (Figs 1–3) were generated by combining 20 to 40 photographs in different focus using the image stacking freeware CombineZM (Hadley 2006). Scanning Electron Microscope (SEM) images of male paratypes of *Strigocis vicosensis* sp. n. and males of *Strigocis bilimeki* were obtained using a LEO 1430VP. Specimens were analyzed under variable pressure (SEM-VP) using a backscattered electrons detector, without prior dehydration or gold covering. The holotype (Figs 1–3) was not dissected. Three male paratypes of the new species and five males *Strigocis bilimeki* were dissected for slide preparation and photographing of terminalia (one of each species shown in Figs 7 and 10, after dissection). Besides these, males of *Strigocis bicornis*, *Strigocis opacicollis* and *Strigocis opalescens* were also dissected for examining their terminalia. Permanent slide preparations were made using a water-soluble mounting media called “Downs’ gel” (polyvinyl lacto-phenol), prepared by mixing 56% saturated aqueous solution of polyvinil alcohol, 22% phenolic acid and 22% lactic acid. Detailed information on its preparation and use are provided by Downs (1943) and Salmon (1947). Examination and photography of slide preparations were made under a Zeiss Axioskop 40 compound microscope equipped with a Canon A640 digital camera.

Terms for external morphology and male terminalia of ciids are explained and discussed by Lopes-Andrade and Lawrence (2005) and Lopes-Andrade (2008). The new species described here was compared to named specimens of all the described *Strigocis*, except for *Strigocis tokunagai*. In the latter case, however, a drawing of an adult male was provided in the original description (Nobuchi 1960).

Ten males (including the holotype) and ten females were measured. Range, mean and standard deviation are given for measurements and ratios. Measurements of antennomeres were taken from the holotype. The following abbreviations are used for measurements and ratios: CL, length of the antennal club; EL, elytral length (median length from base of scutellum to elytral apex); EW, greatest elytral width; FL, length of the antennal funicle; GD, greatest depth of the body; PL, pronotal length along midline; PW, greatest pronotal width; TL, total length (EL+PL; head not included). The ratio GD/EW was taken as an indication of degree of convexity; TL/EW indicates degree of body elongation. The description is based on the holotype, which is a fully pigmented male. Differences among paratypes are given in the section on “Variation”, together with standard measurements and ratios of the type series.

The following acronyms are used in this paper:

LAPCCristiano Lopes-Andrade Private Collection (Viçosa, MG, Brazil)

MZSPMuseu de Zoologia da Universidade de São Paulo (São Paulo, SP, Brazil)

## Taxonomy

### Strigocis vicosensis

Lopes-Andrade sp. n.

urn:lsid:zoobank.org:act:34BA5411-44C2-4D28-B020-0C9F63DB1828

Figs 1 7 

#### Type-locality.

Viçosa, in the state of Minas Gerais, southeastern Brazil (20°45'S, 42°53'W).

#### Etymology.

The specific epithet is an adjective referring to the *terra typica* of the species.

#### Diagnosis.

**Figures 4–7.**
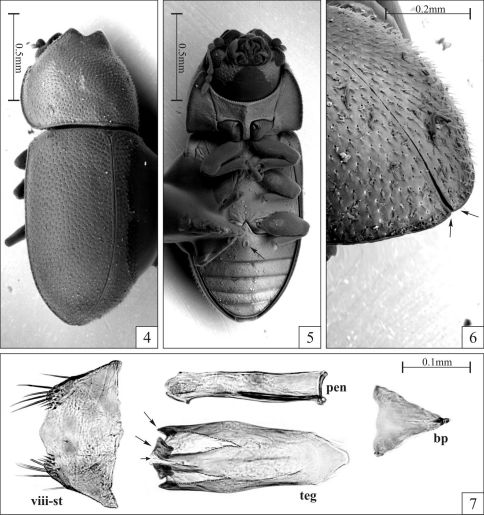
*Strigocis vicosensis* Lopes-Andrade, sp. n., SEM-VP of adult male paratypes (4–6) and slide preparation of a male terminalia (7). **4** Slightly oblique dorsal view **5** Ventral view showing the sex patch at the first abdominal ventrite (arrow) **6** Dorsal view of the posterior portion of the elytra in an oblique position, showing the barely discernible diverging sutural flange (arrows) **7** Male terminalia showing the eighth sternite (pregenital sclerite) and dissected aedeagus with conspicuous subtriangular basal piece, subcylindrical membranous penis and tegmen. Note the emargination at the middle of the tegmen’s apex (small arrow), the two V-shaped concavities (dashed lines), and the two sclerotized tips of each lateral lobe (large arrows). Abbreviations: Eighth sternite (viii-st), basal piece (bp), tegmen (teg) and penis (pen).

Dorsal vestiture consisting of stout yellowish bristles, pronotal punctures coarse and separated from each other by 0.75× to 1× puncture-width, and sutural flange of elytra not diverging near apex. Males have both frontoclypeal ridge and anterior pronotal margin produced forward and emarginated at middle forming two small subtriangular plates (Figs 1, 2, 4, 5). Additionally, the abdominal sex patch of males is small, with around one-fifth the length of the first abdominal ventrite at the longitudinal midline.

#### Description.

Male holotype (Figs 1–3), measurements in mm: TL 1.70; PL 0.55; PW 0.68; EL 1.15; EW 0.73; GD 0.55. Ratios: PL/PW 0.81; EL/EW 1.59; EL/PL 2.09; GD/EW 0.76; TL/EW 2.34. Body elongate, moderately convex; dorsal and ventral surfaces mostly unicolored, almost black, with reddish brown appendages; dorsal vestiture consisting of stout suberect or decumbent yellowish bristles; ventral vestiture consisting of slender decumbent yellowish setae. Head barely seen from above; dorsal surface concave, glabrous; punctation sparse, consisting of course shallow punctures; frontoclypeal ridge slightly raised and produced, its anterior margin with an emargination at middle forming two short subtriangular plates with relatively rounded apices. Eyes coarsely facetted, each one bearing more than 60 ommattidia; greatest eye width 0.10 mm. Antenna (left antenna measured; FL 0.10 mm; CL 0.16 mm; CL/FL 1.63) with length of antennomeres (in mm) as follows: 0.06; 0.05; 0.04; 0.02; 0.01; 0.01; 0.01; 0.05; 0.05; 0.06; sensillifers of the antennal club whitish, conspicuous (seen in magnification of 50×). Pronotum densely punctate, the punctures being coarse and separated from each other by 0.75× to one puncture-width; in between punctures somewhat microreticulate; the stout bristles of the pronotal disc are decumbent, so attached to the surface that they resemble small scales and are more easily visible in lateral view; anterior portion produced forward and almost concealing the head when seen from above; anterior margin bearing an emargination at middle, forming two small subtriangular plates that are slightly larger than those of the head (Figs 1–2, 4–5); anterolateral angles (corners) produced forward and rounded; lateral margins slightly crenulate, almost straight, barely visible from above except for their posterior portions. Scutellum very small, so close to the elytra that makes it barely discernible; posterior margin broadly rounded, so that the entire structure resembles a half-circle; surface bearing small punctures very close to each other, giving a creasy appearance to its surface; basal width 0.07 mm. Hind wings fully developed (macropterous species). Elytra with lateral margins subparallel at basal half, then gradually converging to the apex; punctation single and confused, the punctures being coarser than those on pronotum and closer to each other; bristles similar to those on pronotum, but suberect and a little bit bigger; in between punctures smooth and shiny; sutural flange not diverging near apex (slightly divergent when examined in SEM; see Fig. 6, arrows). Ventral sclerites with most of their surfaces finely granulate. Hypomera unpunctate and bearing a few sparse slender setae. Prosternum biconcave and distinctly tumid at the longitudinal midline, carinate. Each protibial with its outer apical angle expanded forming a small acute tooth; apex bearing a row of spines closest to the inner apical angle. Metaventrite with a few very shallow and coarse punctures, barely discernible; discrimen with one-third the length of the ventrite at the longitudinal midline. Abdominal ventrites bearing several slender setae; first abdominal ventrite twice as long as the second, bearing a small oval margined sex patch, with near one-fifth the length of the ventrite at the longitudinal midline.

#### Male terminalia in paratypes (Fig. 7).

Eighth sternite (Fig. 7) with its posterior margin membranous (tending to collapse during slide preparation), sinuous; corners bearing long setae. Aedeagus (Fig. 7) with near 0.3 mm of length; basal piece large, conspicuous, with two-fifths the length of tegmen; penis subcylindrical, membranous, 0.8× the length of tegmen; tegmen with its posterior portion bearing a narrow emargination at middle (Fig. 7, small arrow) delimiting two lateral lobes, each lobe with a somewhat V-shaped concavity (dashed lines) ending in two sclerotized tips (large arrows).

**Figures 8–10.**
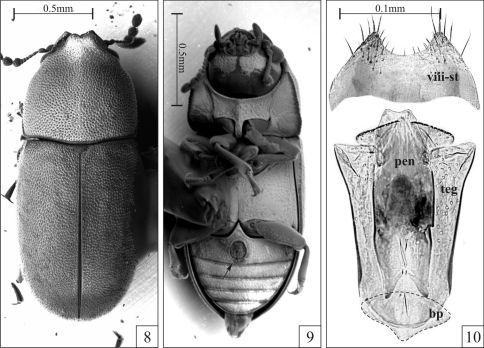
*Strigocis bilimeki* (Reitter) from Cualac (Mexico, Guerrero), SEM-VP of adult males (8–9) and male terminalia (10). **8** Dorsal view **9** Ventral view showing the sex patch at the first abdominal ventrite (arrow) **10** Slide preparation of a male terminalia showing aedeagus and eighth sternite slightly squashed to emphasize their morphology. Note the membranous penis (dotted line) and the small basal piece (dashed line). Abbreviations: Eighth sternite (viii-st), basal piece (bp), tegmen (teg) and penis (pen).

#### Females.

Head with frontoclypeal ridge just slightly sinuous. Anterior margin of pronotum broadly rounded. Vertex of head with sparse suberect stout bristles. First abdominal ventrite devoid of sex patch.

#### Variation.

Males, measurements in mm (n = 10, including the holotype): TL 1.33–1.75 (1.59 ± 0.14); PL 0.50–0.70 (0.60 ± 0.07); PW 0.55–0.75 (0.66 ± 0.06); EL 0.80–1.15 (0.98 ± 0.10); EW 0.63–0.78 (0.70 ± 0.05); GD 0.50–0.60 (0.54 ± 0.04). Ratios: PL/PW 0.81–1.00 (0.92 ± 0.06); EL/EW 1.27–1.59 (1.40 ± 0.09); EL/PL 1.43–2.09 (1.64 ± 0.21); GD/EW 0.73–0.83 (0.77 ± 0.03); TL/EW 2.12–2.34 (2.26 ± 0.08). A few males were almost devoid of secondary sexual characters on frontoclypeal ridge and anterior margin of pronotum. However, the small abdominal sex patch was observed in all available males.

Females, measurements in mm (n = 10): TL 1.30–1.65 (1.52 ± 0.13); PL 0.43–0.63 (0.54 ± 0.06); PW 0.53–0.70 (0.62 ± 0.06); EL 0.83–1.10 (0.99 ± 0.08); EW 0.60–0.75 (0.69 ± 0.06); GD 0.45–0.58 (0.54 ± 0.05). Ratios: PL/PW 0.75–0.96 (0.86 ± 0.06); EL/EW 1.38–1.54 (1.44 ± 0.06); EL/PL 1.60–2.18 (1.85 ± 0.17); GD/EW 0.75–0.81 (0.78 ± 0.02); TL/EW 2.14–2.30 (2.22 ± 0.05).

#### Type series.

Male holotype(MZSP) “BRASIL: MG Viçosa; Campus UFV 18.vi.2006 C.B. Oliveira leg. ex *Phellinus* sp.” “*Strigocis vicosensis* Lopes-Andrade HOLOTYPUS” [printed on red paper]. Paratypes: one male (LAPC), same data as holotype; six females, three males (LAPC) “BRASIL: MG Viçosa; 2° Represa UFV; 20.ii.2006 leg. CB Oliveira”; one female (LAPC) “BRASIL: MG Viçosa 16.ii.2002 leg. C. Lopes-Andrade”; 13 females, five males (LAPC) “BRASIL: MG; Viçosa Campus UFV, 3° represa, próx. supermercado 18.vii.2007; colônia II leg C. B. Oliveira”. All paratypes distinguished labeled “*Strigocis vicosensis* Lopes-Andrade PARATYPUS” [printed on yellow paper].

#### Host fungus.

Possibly a single species of an undetermined *Phellinus* Quél. (Hymenochaetaceae). It’s important to emphasize that only adult ciids were found and they were not observed breeding in the collected basidiomes.

#### Natural history.

All the known specimens were collected in open areas of the Federal University of Viçosa’s campus. It was not found in either forested or urban areas of Viçosa, although ciids were continuously collected there from September 1998 until recently. Adults could not be maintained in laboratory for much longer, which suggests that it colonizes and consumes the basidiomes just after sporulation and before basidiomes’ decaying. Another possibility is that the *Phellinus* sp. is not a host for *Strigocis vicosensis* sp. n., and adults were incidentally collected in the fungus. The latter explanation is supported by the fact that all the other *Strigocis* species are known to feed on fungi in the *Trametes* host-use group and were never found in association with fungi of the *Phellinus* host-use group (Orledge and Reynolds 2005). *Xylographus gibbus* Mellié were found in basidiomes possibly conspecific to the ones inhabited by *Strigocis vicosensis* sp. n., in the same open areas. However, these two ciid species have not been collected together.

#### Comparative notes.

Among the examined *Strigocis*, the most similar to *Strigocis vicosensis* sp. n. is the Mexican *Strigocis bilimeki*. The latter specieshas pronotal and elytral punctation comparatively denser, subtriangular plates of the frontoclypeal ridge in males usually with acute apices and anterior pronotal margin with a short and relatively narrow lamina bearing a small emargination at middle (Fig. 8). Additionally, in *Strigocis bilimeki* the male sex patch in the first abdominal ventrite is very large (Fig. 9, arrow), its diameter being at least 0.55× the length of the sclerite at its longitudinal midline. Male terminalia of both species are similar in form, mainly in the subcylindrical membranous penis (Figs 7, 10) and the shape of the posterior portion of tegmen. However, the whole aedeagus of *Strigocis bilimeki* (Fig. 10) has around 0.8× the length of the one of *Strigocis vicosensis* sp. n., a proportionally smaller basal piece (Fig. 10, dashed lines) and is less sclerotized. Moreover, the posterior margin of the eighth sternite has a broad U-shape emargination (Fig. 10) similar to that of *Strigocis bicornis*, *Strigocis opacicollis* and *Strigocis opalescens*.

*Strigocis opalescens* has a vestiture of minute setae barely visible even in high magnifications (50×), sparse pronotal and elytral punctation, and broad lateral margins of pronotum forming a raised lip. The tegmen of its male terminalia is subquadrate, with a deep U-shaped emargination at middle delimiting two lateral lobes, each lobe bearing a small emargination at apex. *Strigocis bicornis* is a small blackish species with shallow pronotal and elytral punctation, irregular elytral surface with in between punctures finely granulate, and dorsal vestiture of fine setae. Its tegmen is elongate, with a rounded posterior margin bearing a very narrow V-shape emargination at middle. It is similar to the examined named specimens of *Strigocis opacicollis*, in which the elytral margins are not so parallel and elytral punctation is coarser and denser. Additionally, the tegmen in *Strigocis opacicollis* is quite different, with an almost straight posterior margin bearing a small U-shaped emargination at middle. I could not examine any named specimen of *Strigocis tokunagai*, but data and drawing provided by Nobuchi (1960) show that it is similar to *Strigocis vicosensis* sp. n. in the vestiture of yellowish bristles, pronotal and elytral punctation. However, the abdominal sex patch of *Strigocis tokunagai* is described as being large.

## Provisional key to the species of Strigocis Dury

**Table:** 

1	Vestiture dense, especially on elytra, and consisting of short stout bristles	2
1’	Vestiture of somewhat sparse fine setae, conspicuous or not	4
2(1)	Pronotal punctation very dense, punctures separated by less than 0.75? puncture-width; in between punctures granulate, giving a dull appearance to the pronotal surface. Prosternum tumid but not carinate. Mexico and probably southern Arizona	*Strigocis bilimeki* (Reitter)
2’	Pronotal punctures separated by a distance of at least 0.75?; in between punctures microreticulate. Prosternum carinate	3
3(2’)	Male pronotum with apices of the subtriangular plates as separated as the ones of the frontoclypeal ridge. Male with small abdominal sex patch, with around 0.2? the length of the ventrite at the longitudinal midline. Known only from Vicosa (southeastern Brazil)	*Strigocis vicosensis* Lopes-Andrade, sp. n.
3’	Male pronotum with the apices of the subtriangular plates closer than those of the frontoclypeal ridge. Male with large abdominal sex patch. Japan: Hokkaido	*Strigocis tokunagai* (Nobuchi)
4(1’)	Body suboval. Lateral margins of pronotum relatively broad and with a raised lip. Elytral vestiture of barely visible (magnification of 50?) minute setae. Eastern North America	*Strigocis opalescens* (Casey)
4’	Boby subparallel-sided. Lateral margins of pronotum narrow and devoid of raised lip. Elytral vestiture of small but distinct setae	5
5(4’)	Elytral punctation coarse; punctures separated by a distance of one puncture-width or less. Eastern North America	*Strigocis opacicollis* Dury
5’	Elytral punctation fine; punctures separated by a distance of 2? puncture-widths or more. Central and southern Europe	*Strigocis bicornis* (Mellie)

## Discussion

The debate on the morphological limits, and consequently on the species that belong or not to *Strigocis*, is far from being concluded. In its original description, Dury (1917) has not included any other species besides the type-species, *Strigocis opacicollis*. *Strigocis bicornis* and *Strigocis bilimeki* were originally assigned to *Cis* Latreille, *Strigocis opalescens* to *Xestocis* Casey, and *Strigocis tokunagai* to *Ropalodontus* Mellié in their original descriptions. Lawrence (1965) tentatively included *Strigocis bicornis* and *Strigocis tokunagai* in *Sulcacis* Dury possibly due to their spinose protibial apex. Finally, Lawrence (1971) transferred all the four species to *Strigocis* and provided a table of characters for distinguishing it from *Sulcacis*. However, some European taxonomists have considered *Strigocis bicornis* as belonging to *Sulcacis* until recently (see Orledge and Booth 2006 for a brief discussion). The greatest taxonomic problem of the genus, which possibly has led to such instability in literature, is the absence of exclusive characters for defining the taxon.

Lawrence (1971) proposed the spinose protibal apex and the diverging sutural flange of elytral apex as diagnostic to *Strigocis*. At that time, only species of *Strigocis* and *Orthocis* Casey were known to have the latter feature. Diagnostic to *Orthocis* were the rounded protibial apex, devoid of spines, and absence of secondary sexual modifications besides the male sex patch of several species. However, the diverging sutural flange of elytral apex was not observed in *Strigocis vicosensis* sp. n., and it was already observed in *Ennearthron pruinosulus* (Perris), *Wagaicis wagae* (Wankowicz) and *Odontocis denticollis* Nakane and Nobuchi (Lopes-Andrade pers. obs.). The spinose protibial apex is also not exclusive to *Strigocis*, and it may vary among species in a single genus, like in *Cis*. Several species of *Cis* have pronotal and frontoclypeal modifications and body form similar to those of *Strigocis*, as well as spinose protibial apex in which the spines are concealed by long setae. These are the cases for *Cis graecus* Schilsky and species of the *Cis bilamellatus* group (sensu Lopes-Andrade et al. 2009), for instance. In the case of the abovementioned *Cis* species, they are distinguishable from *Strigocis* by their dual elytral punctation.

It is early to take the decision of synonymizing *Strigocis* to *Cis* before a careful taxonomic revision of both groups. Moreover, *Cis* is certainly not a clade (Buder et al. 2008) and remains as the most speciose and morphologically diversified genus of Ciidae, with around 375 described species. Although the matter on the limits of *Strigocis* remains far from being satisfactorily resolved, I prefer to re-evaluate the diagnostic characters of the genus, as follows: (i) protibial apex bearing a row of spines, sometimes concealed by adjacent long setae; (ii) prosternum always biconcave, slightly tumid to carinate; (iii) elytra with both vestiture and punctation single; (iv) frontoclypeal ridge and anterior margin of pronotum in males usually emarginated at middle; (v) sutural flange of the elytral apex diverging near apex in most species (except in *Strigocis vicosensis* sp. n.); (vi) ovipositor with gonostyli well-developed. The latter feature separates *Strigocis* from *Sulcacis*, in which the gonostyli, as far as the whole terminalia, are reduced (Lawrence and Lopes-Andrade 2010).

## Supplementary Material

XML Treatment for Strigocis vicosensis
